# Osteoporosis prevention: Where are the barriers to improvement in French general practitioners? A qualitative study

**DOI:** 10.1371/journal.pone.0219681

**Published:** 2019-07-16

**Authors:** Blandine Merle, Julie Haesebaert, Amélie Bedouet, Loïc Barraud, Marie Flori, Anne-Marie Schott, Christian Dupraz

**Affiliations:** 1 INSERM Unité 1033, Université de Lyon, Lyon, France; 2 Hospices Civils de Lyon, HESPER EA, Université de Lyon, Lyon, France; 3 Collège Universitaire de Médecine Générale, Université de Lyon, Lyon, France; University of British Columbia, CANADA

## Abstract

**Background:**

Osteoporosis prevention, diagnosis and treatment remain suboptimal.

**Objectives:**

We conducted a qualitative study to understand barriers towards care initiation and levers to improve awareness and management of osteoporosis among general practitioners (GPs).

**Methods:**

Semi-structured face-to face interviews were conducted with 16 GPs in the Rhône area of France to explore their knowledge and representations regarding osteoporosis. A thematic analysis of transcripts was performed to identify GPs’ perceptions on osteoporosis diagnosis, prevention, treatment and patients’ expectations.

**Results:**

Interviewed GPs considered osteoporosis far less important than other chronic diseases. They questioned whether osteoporosis was a disease or normal aspect of ageing. They associated osteoporosis with fragility fractures, female sex, menopause, and old age but rarely with male sex. They regarded bone mineral density as the reference diagnostic test, but certain GPs indicated that they had difficulties to interpret the results and to know when to prescribe. Biphosphonates were mentioned as the reference treatment but some GPs expressed distrust about osteoporosis medications. Most of them did not think to screen for osteoporosis risk factors in their patients in a preventive medical approach. They mentioned the lack of time to implement prevention and were expecting clear and pragmatic guidelines, as well as information campaigns in general population to increase awareness on osteoporosis.

**Conclusion:**

GPs tended to underestimate the salience of osteoporosis. Clear recommendations, better awareness of GPs and the general population could improve osteoporosis prevention and treatment.

## Introduction

The burden of osteoporosis (OP) is increasing worldwide [1, 2]. In France, it is estimated that in 2010, 377 000 new osteoporotic fractures occurred, generating costs of nearly 4.8 million euros [3]. The aging population and the increase in life expectancy are likely to increase the prevalence of OP and osteoporotic fractures responsible for a predictable high societal and disease burden [4, 5]. In France, the cost of fractures was estimated to rise by 26% between 2010 and 2025 to reach 6.1 million euros on the basis of expected changes in demographics [3].

Many initiatives have been implemented at the patient and/or healthcare level to improve primary and secondary OP prevention [6, 7]. Guidelines and evidence-based tools are available to help assess patients at increased risk of OP and fracture, and offer an appropriate management [8]. Different barriers limiting the use of evidence-based medicine by physicians have been proposed: insufficient knowledge translation from research to clinicians, gap between guidelines and patients’ reality, heavy workload, lack of knowledge, patient and GPs preferences [9–11]. As first-line care professionals, general practitioners (GPs) represent key actors in the implementation of OP guidelines. However, guidelines are underused; awareness and management of OP are suboptimal leading to less than 20% of patients correctly managed after a low trauma fracture, despite availability of effective medications both worldwide and in France [2, 4, 12, 13]. The reasons are probably multifactorial and related to the patient, but also to the healthcare system. Concerning OP management in France, different clinical trials have demonstrated that improvement is possible [3, 14–17]. Qualitative research is needed to understand how GPs deal with OP and why current evidence on OP management are not integrated in everyday practices [18].

We aimed herein to explore the knowledge and representations of French GPs regarding OP and its prevention, attitudes related to the use of guidelines, perceived barriers to OP care, and suggestions for improvement.

## Methods

A qualitative study was implemented to explore the knowledge and representations of GPs in the Rhône area of France regarding OP care and its prevention. Semi-structured face-to-face interviews were conducted with GPs [19]. This study is part of a larger program aiming to explore knowledge and representations of all end-users of OP guidelines in the French healthcare system [20].

### Participants

All GPs practicing in the Rhône area of France (1,833,000 inhabitants, https://www.insee.fr/fr/statistiques/) at the time of the study were eligible, excluding GPs with additional specific training in geriatrics or rheumatology, to remain representative of general practice. In France GPs are private family physicians, in charge of primary care, practicing alone or in medical centers. Regarding OP, their role is to screen for OP risks factors and, if needed, to prescribe measurement of bone mineral density (BMD). According to results, they can initiate a treatment or address the patient to a second-line physician (rheumatologist) to do it. The long-term follow-up can be undertaken by the GP or rheumatologist according to the patient preference.

We approached GPs by a phone call using a random selection process among the list of 1450 telephone numbers of GPs practicing in the Rhône area in 2014. The objectives and methods of the study were explained and they were invited to participate. GPs accepting to participate received written information from the investigation center on the study objectives, interview process, and confidentiality of the data. Participation was effective only after the written informed consent was obtained. Recruitment ceased when data saturation was reached i.e. successive interviews did not generate new information, but a minimum of 15 interviews was planned.

The study was conducted in accordance with the World Medical Association Declaration of Helsinki. It was submitted and approved by the French ethics committee of the university hospital of Saint-Etienne, France, Institutional Review Board: IORG0007394 (Ref: IRBN102014/CHUSTE), and by the national French data protection authority (CNIL).

### Procedures and data collection

We conducted a descriptive qualitative study with GPs, to gain practical answers to questions concerning their perception and usual management of OP [21] [22]. Individual face-to-face semi-structured interviews were conducted by AB (resident in family practice trained for semi-structured interviews), at the GP office. The interview guide had been developed by the authors based on a literature review. It had been enriched and validated by the study steering group composed of GPs, public health researchers and rheumatologists, and through a pilot interview. The interview guide allowed both to make sure to address all the preset themes and to prompt GPs in case the answer was not sufficiently developed. Each discussion started with a word association task by the question: *‘If I say osteoporosis*, *what words come to mind’*. Five domains were then covered: OP representation, diagnosis, management, prevention, projection of patients’ ideas on OP (Table 1). Questions remained deliberately broad and open to allow the emergence of new themes. For each topic, representations and physicians’ knowledge, difficulties, and suggestions for care improvement were investigated. Interviews were audio-recorded and non-verbal interactions were noted. Information about demographic characteristics and type of practice was collected before the interview.

**Table 1:** Domains developed in the interview.

Domains	Questions
**Disease representation**	- The first 3 words that come to your mind when I say “osteoporosis”? - Profile of an osteoporotic patient?
**Diagnosis**	- Who is concerned? How to diagnose OP? - Which difficulties do you meet? - What are your expectations for improvement?
**Management**	- Which steps in the therapeutic management ? - Which treatment? What for?
**Prevention**	- OP prevention: useful? for whom? - What are your expectations to improve prevention?
**Patient knowledge**	- What do you think patients know about OP? - What are the expectations of the patients concerning OP prevention and care?

OP: osteoporosis

### Data analysis

Analyzed data comprised audio-recording and field notes of the interview. The audio-recording of interviews have been comprehensively transcribed by AB. An inductive thematic analysis of the transcribed interviews was performed by AB using NVivo 10 software (QSR International Pty Ltd, Doncaster, Australia) [21]. CD and LB reviewed and validated coding generated by AB.

Each idea was coded in order to consolidate occurrences. The occurrences were grouped into main themes, refined in a continuous and iterative process as new ideas emerged. A cross-sectional analysis was then performed. Once the main themes emerging from the interviews were defined, a return to the transcripts was done to select quotes to illustrate each theme. All data were handled confidentially. The interviews and transcription were conducted in French; the quotes mentioned in this paper were translated into English and reviewed by a native English scientific writer for consistency.

## Results

### Participants

A total of 77 GPs were contacted by telephone. Sixty refused to participate; the main reasons were lack of time, no interest in participating and personal reasons. One GP specialized in geriatrics was excluded. Sixteen agreed to participate and provided written informed consent. Interviews were held between June 2014 and January 2015. They lasted a mean 26 minutes (range: 15 to 41 minutes). The socio-demographic characteristics of the GPs involved are described in Table 2, the verbatim quotes are reported in Table 3, and the main results are summarized in Table 4.

**Table 2:** Sociodemographic and practice characteristics of the participants (n = 16).

**Sex:** women, n (%)	5 (31)
**Age** (Mean ± sd):	55 ± 10
- 31–50 years old: n (%)	4 (25)
- 51–70 years old: n (%)	12 (75)
**Type of practice:** n (%)	
- single	6 (38)
- group	10 (62)
**Practice in urban area:** n (%)	14 (87)
**Number of years of practice:** (Mean ± sd)	24 ± 10
**Visit to institutionalized elderly patients:** n (%)	
- no	9 (56)
- yes	7 (44)
**Training on OP through:** n (%)	
- continuous medical education	15 (94)
- internet	8 (50)
- medical press	13 (81)
- medical sales representatives	8 (50)

Results are expressed as n (%) or Mean ± sd

**Table 3:** Quotes from the quote interviews of GPs.

n°	Quote
**1**	GP01: ‘an inactive elderly person who stays at home, infrequently going outside, with a vitamin D deficiency, is he/she with a greater risk of fracture? I have not noticed…’
**2**	GP13:’first I have to accept, and I haven’t done it yet, that OP is something other than normal progression.’
**3**	GP12: ‘It exists in men, for sure, but I have never seen it; that's why for me it is not too masculine.’
**4**	GP14: ‘A hip fracture in an elderly person, it can be very serious, it can be a way to decompensate many things… and finally to die!’
**5**	GP15: ‘Post menopausal women, who complain about diffuse pain, we program a bone densitometry;… sometimes it's a little bit by chance; OP diagnosis, it's a bit empirical.’
**6**	GP11: ‘… we do not necessarily have a correlation between fracture frequency and BMD results, therefore what is right? Do we have the right marker?
**7**	GP06:’ …difficulties in interpreting the figures, T score, Z scores …and FRAX: I calculate it on the internet, I have a percentage, but I do not know what to do? It is said "it's great you have a percentage of risk of …" yes, but so what? ‘
**8**	GP10: ‘what would be great is to have a mass screening, like a mammography.’
**9**	GP04: ‘We should consider OP, because there is an aging population and we have to prevent degradation of health in the very elderly.’
**10**	GP03: ‘when it is complicated when women have a fracture while they are already on a treatment, in which case I send them to a rheumatologist.’
**11**	GP15: ‘It is our role to explain…If they (patients) do not really understand what is OP, I think that they will not accept treatment. ‘
**12**	GP08: ‘…To know with which one to start, when to switch, because we will have a treatment for 20 years, it is not easy… we did not really have a lot of information on it, and it's not so easy to prescribe.’
**13**	GP16: ‘…side effects! for a benefit that has not really been proven! ‘…, I think there's more osteonecrosis of the jaw with the long-lasting treatment’
**14**	GP 11: ‘we are always told, at least by the labs, the percentage of reduction in events without really talking about the number of events without treatment.’
**15**	GP 08: ‘people do not feel sick, so taking a medicine when you do not feel sick is always difficult to accept.’
**16**	GP05: ‘I do not know if I am very convinced and convincing … it is also the problem, if the physician is convinced, patients will be more convinced; sometimes I am, and sometimes less, patients may feel it.’
**17**	GP 05: ‘I will not speak of OP systematically, far from it! because I already have so many other things to talk about, diabetes, and smoking, and alcohol, and …’
**18**	GP16: ‘ … it is difficult to say from what age, from what point is it really is a disease.’
**19**	GP09 : ‘… to make the patient understand, via the media for example, that OP is a factor inducing a reduction in the quality of life, and even of lifespan, I believe that's what's missing…’
**20**	GP02: ‘when people's attention is attracted by the media … suddenly, we can convey messages more easily.’
**21**	GP16: ‘there are too many messages and so, people are no longer sufficiently concerned.’
**22**	GP15: ‘they become aware of OP when they fracture; before, it's not important.’
**23**	GP13: ‘they are afraid of aging… one falls, fractures, then we are in bedridden, then the hospital, and then the person dies! It's aging … the disease itself, called osteoporosis or natural aging, I do not think they distinguish!’
**24**	GP01: ‘they are waiting for me to put them in the best possible situation to age well!’

**Table 4:** Main results.

Domains	Results
**Disease representation**	- OP is considered as a minor disease and infrequent - OP concerns mainly elderly menopausal women
**Diagnosis**	- Difficulties to identify at-risk patients - BMD is the gold standard for the diagnostic
**Management and treatment**	- Necessity of practical and easy-to-use guidelines - Bisphosphonates are the reference treatment - GPS not familiar enough with the available medications and aware of the deleterious side-effects and compliance problems with some medication
**Prevention**	- More information and prevention should be done in general population - Prevention is important but there is a lack of time
**Patient knowledge**	- Most Patients have never heard of OP - Patients often do not feel concerned

OP: osteoporosis

### General representations of Osteoporosis

In the word association task, GPs first associated OP with fracture, female sex, aging, and family history. They defined OP as increased bone fragility due to a loss of calcium, concerning mainly post-menopausal women and the elderly, and leading to a high risk of fracture. However, the association between OP, vitamin D/calcium deficiency and fracture was sometimes questioned (*quote 1*). Whether OP is a true disease or natural bone deterioration was questioned. GPs expressed difficulties to define a clear limit between normal and pathological bone aging and did not know when to consider bone fragility as a disease to treat (*quote 2*).

The profile of the osteoporotic patient was defined by the following characteristics: old age, female sex, low body mass index, familial and/or personal history of OP or fracture, long-term corticosteroid therapy. A sedentary lifestyle, tobacco smoking were also mentioned as potential risk factors. They all spontaneously talked about OP in women, most GPs did not think of OP in men, as it was considered to be infrequent and often minimized (*quote 3*). Concerning symptoms and consequences, GPs reported that OP was responsible for fractures affecting mostly the femoral neck, wrist and spine, and was sometimes associated with pain. It was usually the fracture that led to investigate OP. Most physicians were aware that the first fragility fracture led to an increased risk of future fracture with a high associated morbidity and mortality (*quote 4*).

### Osteoporosis diagnosis

There was a consensus to say that Bone Mineral Density (BMD) assessment is the gold standard to confirm the diagnosis and follow patients with OP or osteopenia. OP investigation was usually engaged for patients presenting known OP risk factors. But several interviewees considered those risk-factors as non-OP specific, inducing difficulties to think about screening for OP in these patients (*quote 5*). Occasionally, BMD assessment was a request from the patient. GPs thought that BMD measurement should be done preferentially by a rheumatologist or in specialized centers. However, some GPs experienced difficulties in investigating OP: they felt unease to correctly identify at-risk patients and when to prescribe a BMD. They sometimes expressed their lack of confidence in BMD results and did not feel at ease to interpret the results of BMD or the WHO fracture risk assessment tool (FRAX) (*quotes 6 and 7*). To improve screening and diagnosis, some GPs suggested to implement a systematic OP screening as it is done for breast cancer, with a BMD test covered by the national healthcare insurance (*quote 8*).

### Osteoporosis management and treatment

Although they agreed that OP was potentially a debilitating condition, concern about it varied greatly among GPs. Other chronic conditions such as diabetes or cardiovascular diseases were generally considered as more serious health issues. However, with population aging, some GPs were convinced of the importance of a more appropriate OP management (*quote 9*). They regretted the absence of consensus about the professional in charge of OP in France (GPs, gynecologists or rheumatologists), leading to a lack of care or, conversely, to redundant drug prescription. GPs quite often referred their OP patients to rheumatologists; as they do not see many OP patients, several did not feel sufficiently confident and preferred to entrust the entire follow-up to a rheumatologist, particularly the treatment prescription for which they did not feel sufficiently informed (*quote 10*).

Treatment prescription was one step in a multistep process of OP care. GPs had to validate the diagnosis, give nutrition, lifestyle and fall prevention advice, and explain the benefits and harms of treatments (*quote 11*). They prescribed OP medication for no more than 5 years, according to guidelines but also to the patient personal history and wishes. The objectives were to improve bone mineral density and prevent fragility fractures in order to improve the future quality of life. Vitamin D/ calcium supplementation was considered as the first step of OP treatment, usually prescribed as soon as osteopenia was detected. Among the various classes of OP medication mentioned, bisphosphonates were the most cited. GPs did not feel sufficiently informed about the pharmacological treatments, rendering sometimes the prescription difficult (*quote 12*). However, several were skeptical about the efficacy and long-term safety of some medications. They were aware of their deleterious side-effects and their strict dosing recommendations which can be challenging for patients, explaining their reluctance to prescribe (*quote 13*). They often mistrust pharmaceutical companies (*quote 14*).

Medication non-adherence was a great problem and GPs often felt powerless. They thought that a great barrier to patient adherence was their mistrust in treatments, poly-medication, the constraints and intolerance problems with calcium and bisphosphonates for example. In addition, they said that patients do not see any need to take a treatment for an illness they do not suffer from (*quote 15*).

Finally, GPs were expecting clear scientific messages for themselves, as well as relevant and pragmatic guidelines to correctly manage OP; but they also needed to be themselves convinced of the relevance of OP care (*quote 16*).

Some GPs recognized that they needed training on OP management, even if they all declared to seek for information through different ways, mostly medical training and specialized press (Table 2). Compared to other chronic conditions, some clinicians thought that training on OP is too infrequent.

#### Osteoporosis prevention

They all agreed with the idea of prevention in general, but they were divided about OP prevention, some of them considering it was essential while others thought it was not a priority for a condition considered much less serious than diabetes or cholesterol (*quote 17*). The main issues raised were to know when to start prevention and when to talk about (*quote 18*), and that this was further complicated by the short time allowed for the consultation. Prevention usually included education, screening, and initiation of supplementation or treatment: GPs informed the patient about the importance of a balanced diet and physical activity, screened for risk factors and, if needed, prescribed a BMD; vitamin D/calcium supplementation or a pharmacological treatment will be initiated if required.

To improve OP prevention, they expected more information on OP and its prevention to be given to the general population to create a greater awareness of OP and its consequences (*quote 19*). It should be done by actors other than GPs, i.e. media, national health system, school. If patients were better informed, they would feel more concerned and able to discuss and collaborate with their GP (*quote 20*). Conversely, a few of them thought that people were overwhelmed with prevention campaigns and questioned the effectiveness of public messages, which sometimes lead to misconceptions (*quote 21*).

#### Patients’ knowledge and expectations

The GPs interviewed thought that their patients had poor knowledge of OP, and generally did not care much about it but rather more about the fragility fracture that was considered as the real health problem. They felt that patients regarded OP as serious after a first fracture event (*quote 22*) and generally associated it with the fear of aging and dependence (*quote 23*). Men often knew nothing about it; women were generally better informed, through internet, magazines and television programs mostly designed for senior women, with misconceptions sometimes. People often mixed up OP with osteoarthritis, imagined that physical activity was a risk factor for fracture and were suspicious about treatments. Finally, most respondents thought that patients expected from their GP information about the disease, prevention advice, and the best possible care in their approach to “age well” (*quote 24*).

## Discussion

The objective of the present study was to explore the views of French GPs on OP management and to identify the potential barriers impacting OP care. Most interviewees did not feel too concerned with OP, which was considered far less salient than other chronic diseases and not life-threatening. These findings are in line with the existing literature [18, 23]. OP was associated by GPs with fragility fractures, female sex and old age. BMD was considered as the gold standard for diagnosis and bisphosphonates as the reference treatment. Some GPs questioned the efficacy and side effects of OP medications, and raised the possibility that OP could rather be a natural aging process than a disease. Both considerations hinder OP diagnosis and management, and are also expressed in the general population, both in France and elsewhere [18, 20, 24, 25].

On the whole, OP remains for most interviewees a debilitating condition concerning mainly older, post-menopausal women. GPs did not consider men and younger women for primary or secondary prevention despite the existence of reports that OP is responsible for morbidity and high mortality in men [23, 26]. As the relationship between fragility fractures and OP was often underestimated by GPs, many fractured patients are not screened for OP reflecting the existing post-fracture care gap, both globally and in France [12, 13, 18, 27, 28]. OP prevention was not a priority and many participants reported that they rarely screened patients for OP risk factors in a preventive approach. It will be crucial to increase GPs’ knowledge and concern for OP, as awareness is the first step to guidelines implementation in clinical practice [29].

The investigation for OP was in most cases initiated by the GP and it usually included a BMD measurement. In the present study, GPs expressed uncertainty in interpreting BMD results, and had a greater confidence in BMD reports from rheumatologists rather than from radiologists who may not consider associated risk factors, as reported elsewhere [30]. They suggested the implementation of a mass screening for OP including the estimation of patient risk factors and a BMD measurement if necessary, using a systematic approach in the general population as done in France, for instance, for breast cancer screening. Another point raised by GPs as a limitation to optimal OP management, was to clearly define which physician is in charge of OP care: in France, the GP is the main provider of OP care, but other specialties (i.e. rheumatologists, gynecologists) may also be in charge of it [31]. Furthermore, orthopedic surgeons and emergency physicians are the first healthcare providers to meet patients with fragility fractures, but they seldom send them to their GP for OP screening, missing an opportunity to initiate an appropriate follow-up [32, 33]. GPs expressed the need to clarify the role of each physician and to improve communication and cooperation between all stakeholders to ensure a continuity of care.

New models of care such as Fracture Liaison Services (FLS) or interventions based on a central coordinator, directed to the healthcare providers, patients or both, have been proposed to ensure the continuity of care, from the fracture care to the management of the underlying OP [15–17, 34]. Such models, aimed at increasing awareness and changing the behaviors of clinicians and/or patients, showed significant efficacy to improve OP care. These strategies often require deep organizational and behavior changes as well as human and financial resources that may not be available, preventing their generalization. However, to estimate the osteoporotic fracture risk of a patient through an in-depth anamnesis including the patient’s personal and family history and illness experience, should be the first step of a patient centered care. An exchange between the patient and the GP, focusing on the patient knowledge, needs and preferences, perceived barriers to medication may stimulate his/her involvement in a shared decision process; a patient-physician partnership based on trust and patient’s autonomy should improve patient adherence to treatment and prevention advice, and ultimately improve patient outcome [35].

Several difficulties related to patients have been reported by the GPs for the initiation of OP treatment: over-medication in elderly patients, non-adherence due to a lack of concern, and suspicion about treatment side effects; this skepticism mirrors the distrust toward OP medications expressed in the general population that we reported recently [20]. Treatment non-adherence was perceived as the main issue, because poor medication adherence reduces the benefit of screening and prevention [18, 36–38]. Compliance and persistence are associated with appropriate perception of the condition and could be improved if GPs were themselves convinced and persuasive with their patients about the benefit of prevention and medication [34]. The communication between GPs and patients is essential: as primary care provider, the GP should explain without generating anxiety that OP is a serious health condition for which the patient has the possibility to act [36, 39]. GPs evoked the lack of time to implement prevention in their daily clinical practice, and supported the idea that more information should be given to general population by committed national health services through adapted tools or dedicated persons. Educating and empowering patients would enable them to discuss OP with their GPs and may prompt the clinician to consider this condition and its management easier [39].

Other barriers related to the gap between everyday practice and guidelines have been highlighted as responsible for the inadequate use of guidelines. Interviewees reported they were facing patients and clinical situations that often did not fit to published guidelines, considered as non-pragmatic. Recommendations deal with the management of patients after the validation of an OP diagnosis, and consider OP as a single pathology without other associated condition. But GPs faced aged and often poly-medicated patients with several symptoms and co-morbidities, making OP diagnosis and management sometimes difficult. Due to the nature of primary care, they felt overloaded with information and recommendations on a variety of pathologies they have to deal with, and expressed the need for synthetic, clear and pragmatic guidelines relevant to daily situations. GPs mentioned the lack of collaboration and a growing gap between everyday practice and research; they are waiting for a better level of evidence and a clearer description of the applicability of research evidence to primary care to encourage them to change their behavior [10, 11, 40]. Knowing the barriers to OP guidelines should help develop new recommendations and tools relying on clinicians’ everyday experience and needs, in a ‘bottom-up approach’ [10, 11]. Models of care tailored to clinicians’ practice will ensure their acceptation and implementation [41].

The study does, however, have certain limitations. For instance, only a few of the GPs approached accepted to participate suggesting that the respondents may have a greater-than-average interest in OP precluding the generalization of the results. Although it was explained before the interview that it was not an assessment of professional practices, some GPs may have considered that it was a test of knowledge. During interviews, open-ended questions and the greatest possible neutrality of reformulations were used to ensure that there was no judgment on what was said, to encourage the emergence of occurrences without inducing them.

## Conclusion

The present study highlighted the necessity to raise GPs’ awareness on OP, and alert them on male OP. It will be essential to increase their knowledge on OP care and medication. More qualitative studies are needed to clearly identify barriers to OP management for GPs, but also in all the stakeholders of the healthcare system (gynecologists, rheumatologists, emergency physicians, orthopedic surgeons, radiologists). These will allow the development of pragmatic and easy-to-use guidelines or training aligned with physicians’ needs and support them to implement evidence-based practices regarding OP care. Interventions directed to overcome these barriers should be associated with education of general population. Implementation studies are needed to assess the real-life impact of these interventions. With the ageing of the population, improving OP management in an evidence-based medicine process will be a challenge for the future.

## Supporting information

S1 File Interview guide (French version). (DOCX)

S2 File Interview guide (English version). (DOCX)
