# Supplementary material for: Osteoporosis prevention: Where are the barriers to improvement in French general practitioners? A qualitative study
Source: PLoS One. 2019 Jul 16;14(7):e0219681. doi: 10.1371/journal.pone.0219681 (PMC6634405; doi:10.1371/journal.pone.0219681)
Supplement: S1 File — (DOCX) [file pone.0219681.s001.docx]

**QUALIOP-MG : GUIDE D’ENTRETIEN INDIVIDUEL**

**Représentation de la maladie:**

1- « associations de mots » : quels sont les 3 premiers mots qui vous viennent à l’esprit lorsqu’on vous parle d’OP. Pourquoi ces 3 mots ?

2**-Remémorez-vous le vécu de votre dernière patiente** pour sa prise en charge de l’ostéoporose (OP), **racontez** son histoire et sa prise en charge.

3-Quel est pour vous **le profil du patient ostéoporotique ?** (aide : âge, corpulence, antécédents, facteurs de risque, contexte diagnostic, relancer sur « et chez l’homme ?)

**Diagnostic :**

4-Comment faites-vous le **diagnostic d’OP ?**  (Aide : qui diagnostiquer ? quels examens réaliser ? Quelle est la définition de l’ostéoporose? )

5-Quelles sont **vos difficultés rencontrées pour poser le diagnostic ?** (y penser ? prendre le temps ? manque de connaissance ?)

6-Quelles sont vos **attentes en** vue d’une amélioration**pour vous aider à faire le diagnostic**?

**Prise en charge thérapeutique :**

7-Quelles sont selon vous les étapes de la **prise en charge thérapeutique de l’ostéoporose** ? (Aide : Après résultat examen ? En prévention après une fracture ? Après avis spécialiste ? Comment peser le rapport bénéfice/risque ? Quel traitement choisir ?)

8-Quel est **l’objectif du traitement** selon vous ?

9-Quelles sont vos **attentes** pour une meilleure prise en charge thérapeutique ? (aide : formation ? outils ? documents ?)

10- Comment définiriez-vous une bonne observance ? A votre avis quels sont les freins à une bonne observance du traitement  (Effets indésirables ?) ? Et au contraire quels sont les facilitateurs ?

**Prévention**

11- La prévention de l’OP, qu’en pensez-vous? (Parlez-vous de l’OP à vos patients femmes? et hommes? Si oui comment ? Si non  pourquoi ? Manque de temps, pas trop informé sur OP, pas crucial…)

12- Que pensez-vous que les **patients attendent** de leur médecin en matière de prévention de l’OP ?

13- Quelles sont **vos attentes** en matière de prévention ? (Des campagnes de sensibilisation grand public ? une info par les services de santé auprès des seniors?)

**Connaissances des patients**

14- Que pensez-vous que **les patients connaissent de l’OP** ? (représentation de l’OP chez les patients atteints ? et naïfs ? A quel niveau de gravité évaluent-ils l’OP ? comment fait-on le diagnostic ? Comment traite-t-on ?)

15- Que pensez-vous que **les patients attendent** de leur médecin pour la prise en charge de l’OP ?
