# Supplementary material for: Osteoporosis prevention: Where are the barriers to improvement in French general practitioners? A qualitative study
Source: PLoS One. 2019 Jul 16;14(7):e0219681. doi: 10.1371/journal.pone.0219681 (PMC6634405; doi:10.1371/journal.pone.0219681)
Supplement: S2 File — (DOCX) [file pone.0219681.s002.docx]

**QUALIOP-GP: INDIVIDUAL INTERVIEW GUIDE**

**Representation of the disease**

1- "word association task": what are the first 3 words that come to your mind when I say “osteoporosis”? Why these 3 words?

2- **Remind yourself of your last patient’s** **experience** concerning the management of osteoporosis (OP), **tell** her story and her care.

3-What is **the typical profile of the osteoporotic patient** for you? (i.e. age, corpulence, familiar history, risk factors, context of diagnosis; chase up with “and in men ?”)

**Diagnosis:**

4-How do you make the **diagnosis of OP**? (i.e. who is concerned? which tests to undertake? what is the definition of OP?)

5-What are **your difficulties to make the diagnosis**? (i.e. to think about OP? To take the time? A lack of knowledge?)
6-What are your **expectations** to help you **to make the diagnosis**?

**Therapeutic management**

7-According to you, what are the steps in **the therapeutic management of OP**? (i.e. after the result of an examination? prevention after a fracture? After specialist advice? And, how to assess the benefit / risk ratio? Which treatment to choose?)

8-What do you believe is the **purpose of the treatment**?

9-What are your **expectations** for a better management of OP? (i.e. training? tools? documents?)

10- How would you define good compliance? In your opinion, what are the barriers to good compliance (Side effects?)? Conversely, what are the facilitators?

**Prevention**11- What do you think about OP prevention? (Do you discuss OP with your female patients? And with your male patients? If so, how? If not, why? Lack of time, lack of knowledge about OP, not crucial ...)

12- What do you think the **patients expect** from their doctor concerning OP prevention?

13- What are **your expectations** in terms of OP prevention? (Awareness campaigns in the general population? Information by the health services directed to the at-risk population?)

**Patient knowledge**

14- What do you think the **patients know about OP**? (Representation of OP in affected patients? And in naive patients? How serious do they consider OP to be? Their knowledge about diagnosis? About treatment?)

15- What do you think **the patients expect from their doctor** for OP management?
